# Area-aggregated assessments of perceived environmental attributes may overcome single-source bias in studies of green environments and health: results from a cross-sectional survey in southern Sweden

**DOI:** 10.1186/1476-069X-10-4

**Published:** 2011-01-17

**Authors:** Kim de Jong, Maria Albin, Erik Skärbäck, Patrik Grahn, John Wadbro, Juan Merlo, Jonas Björk

**Affiliations:** 1 Division of Occupational and Environmental Medicine, Lund University, Lund, Sweden; 2 Division of Occupational and Environmental Medicine, Lund University Hospital, Lund, Sweden; 3 Swedish University of Agricultural Sciences, Alnarp, Sweden; 4 Unit for Social Epidemiology, Dept. Clin. Sci. Faculty of Medicine, Lund University, Malmö, Sweden; 5 Competence Centre for Clinical Research, Lund University Hospital, Lund, Sweden

## Abstract

**Background:**

Most studies assessing health effects of neighborhood characteristics either use self-reports or objective assessments of the environment, the latter often based on Geographical Information Systems (GIS). While objective measures require detailed landscape data, self-assessments may yield confounded results. In this study we demonstrate how self-assessments of green neighborhood environments aggregated to narrow area units may serve as an appealing compromise between objective measures and individual self-assessments.

**Methods:**

The study uses cross-sectional data (N = 24,847) from a public health survey conducted in the county of Scania, southern Sweden, in 2008 and validates the Scania Green Score (SGS), a new index comprising five self-reported green neighborhood qualities (Culture, Lush, Serene, Spacious and Wild). The same qualities were also assessed objectively using landscape data and GIS. A multilevel (ecometric) model was used to aggregate individual self-reports to assessments of perceived green environmental attributes for areas of 1,000 square meters. We assessed convergent and concurrent validity for self-assessments of the five items separately and for the sum score, individually and area-aggregated.

**Results:**

Correlations between the index scores based on self-assessments and the corresponding objective assessments were clearly present, indicating convergent validity, but the agreement was low. The correlation was even more evident for the area-aggregated SGS. All three scores (individual SGS, area-aggregated SGS and GIS index score) were associated with neighborhood satisfaction, indicating concurrent validity. However, while individual SGS was associated with vitality, this association was not present for aggregated SGS and the GIS-index score, suggesting confounding (single-source bias) when individual SGS was used.

**Conclusions:**

Perceived and objectively assessed qualities of the green neighborhood environment correlate but do not agree. An index score based on self-reports but aggregated to narrow area units can be a valid approach to assess perceived green neighborhood qualities in settings where objective assessments are not possible or feasible.

## Background

The influence of natural environments, green spaces, on health and well-being has received considerable attention from several disciplines, including environmental psychology, landscape planning and epidemiology [1-7]. Exposure to green neighborhood environments is thought to yield direct health effects through restoration of stress and attentional fatigue [1,2,5,8-10], while health could indirectly benefit via increased levels of social interaction and physical activity [4,11]. Most studies which have assessed neighborhood environments have either used objective measures [3,4,6,12-14] or self-assessments [15,16]. Finding objective assessments that fully cover the health promoting aspects of the physical attributes of green neighborhood environments is a challenging task that requires access to detailed landscape data. Furthermore, perceived environmental attributes may have health promoting effects that objective measures do not capture [17,18]. On the other hand, individual self-reported qualities of the green environment are likely to be influenced also by socio-demographic and personal characteristics, as well as by health status [19], and may therefore yield confounded results (single-source bias), especially if used in cross-sectional settings.

One way to overcome single-source bias is to use aggregated self-reports. Extending data from individual self-reports to area-level assessments of the perceived environment creates a hierarchy in the data which can be handled with multilevel models: a methodology that has been denominated as ecometrics [20,21]. Multilevel models have been used previously in studies examining associations between green environments and health [3,4,14] and physical activity [22]. Such models have also been used in studies to validate specific environmental measures consisting of several individual items, e.g. in relation to criminality [21], neighborhood walkability [23] or to cardiovascular disease risk [20], and to assess contextual phenomena of health variation [24,25].

The overall aim of the present study was to assess if area-aggregated assessments of perceived qualities of green neighborhood environments, as a compromise between objective measures and individual assessments, may overcome single-source bias when investigating associations between self-assessed environmental measures and concurrent health status and health-related behavior on individual level. The study uses cross-sectional data from a public health survey conducted in the county of Scania, southern Sweden, in 2008 and also validates the Scania Green Score (SGS), a new index comprising five self-reported green neighborhood qualities, in relation to objective GIS-based assessments.

## Methods

### Survey participants

The total study population consisted of all people of age 18 to 80 registered as inhabitants of the Scania county, Southern Sweden on 30 June 2008 (N = 899,923). The population was stratified by gender and geographical area, resulting in 2 × 71 = 142 strata. Random sampling from the population registry was used, with an approximately equal number of individuals selected in each stratum. An extensive general health questionnaire was mailed to the 52,142 selected individuals in September-October 2008. There was an opportunity to respond via the web. After three reminders, a total of 28,198 participants had responded (54.1%). The response rate was lower among males, age group 18-34, participants with only compulsory education, low income and those born outside Sweden [26]. Available landscape data (see below) did not permit objective assessment of green neighborhood qualities in the inner city areas of the four major cities (Malmö, Helsingborg, Lund and Kristianstad) in Scania and we therefore had to exclude participants from these areas (N = 3,169). Another 182 participants did not have a valid residential address in Scania and were excluded for this reason, resulting in a final sample of 24,847 participants (table 1).

**Table 1:** Basic characteristics for 24,847 participants of the public health survey in suburban and more rural areas of the Scania region in Southern Sweden (2008).

Subgroup		N_a_	All	Objectively assessed number of green qualities within 300 m		
				0	1	2-4

			N = 24,847	N = 14,350	N = 6,363	N = 4,134

Sex		24,847				

	Females		54.8	55.2	54.0	54.4

	Males		45.2	44.8	46.0	45.6

Age		24,847				

	18-34		20.0	21.6	20.0	14.5

	35-49		26.3	26.3	26.3	25.9

	50-64		30.3	28.7	30.9	34.9

	64-80		23.4	23.4	22.7	24.7

Educational level		22,400				

	Primary and/or secondary school		28.7	29.0	28.3	28.4

	2-4 years gymnasium or professional school		36.4	36.3	37.0	35.7

	University		34.9	34.6	34.6	36.2

Problems with paying bills		24,291				

	Yes, at least every second month		7.1	7.6	6.7	6.3

	No, never or once		92.9	92.4	93.3	93.7

Country of origin		24,847				

	Other		14.0	17.3	11.0	7.2

	Sweden		86.0	82.7	89.0	92.8

Type of residence		24,294				

	Flat or student room		37.6	46.8	31.2	15.3

	Own house		62.4	53.2	68.8	84.7

N_a _Number of answers

Additionally, descriptive information was stratified according to the total number of objectively assessed green qualities (0, 1 or 2-4) within 300 meter from participants' residences. All frequencies are given as percentages if not otherwise stated.

### Assessment of green qualities

Based on interview studies among lay-people, carried out between 1995 and 2005, eight perceived qualities of green neighborhood environments have been indentified [5,27]. These have been implemented as indicators for impact assessment in planning for housing and infrastructure development projects in Scania [28]. Landscape data and GIS allowed us to assess objectively the availability (yes/no) in residential neighborhoods for five of these green qualities: Culture, Lush, Serene, Spacious and Wild (see Additional file 1: appendix 1 for original descriptions and GIS inclusion criteria). Previous research (2004) in the present population showed that objectively assessed availability of these qualities was positively associated with neighborhood satisfaction and physical activity [12]. GIS assessments of the five green qualities (Additional file 1: appendix 1) were based on the National Land Survey of Sweden (Lantmäteriet) that is part of EU program CORINE [29] in which the land and vegetation cover was mapped into approximately 58 classes. Additionally regional GIS databases from the County Administrative Board of Scania were used.

Geocodes for the residential addresses of survey participants, in combination with the landscape databases were used to assess the availability of the five green qualities within 100, 300 and 500 meter from the residence. Presented results refer to the GIS-based objective assessments within 300 meter from the residence unless otherwise stated.

Perceived availability of either one of the green qualities was measured in the survey by asking participants to score their agreement with availability of Culture, Lush, Serene, Spacious and Wild within 5-10 minutes walking distance from their residence (see Additional file 2: appendix 2 for phrasing of the questions). In order to compare self-assessments with the binary GIS assessments, we dichotomized the self-reports by taking 'totally not agreeing' and 'not agreeing' together as perceived 'absence' of the green neighborhood quality and 'agreeing' and 'totally agreeing' were taken together as perceived 'availability' of the quality. SGS was calculated as the sum of the dichotomized positive assessments (range 0 - 5). Missing answers and 'do not know' (10% of all assessments) for the individual items were counted as zero in the index score, but excluded in the assessment of agreement of the five individual qualities. For comparison, a corresponding GIS index score was calculated as the sum of the five objectively assessed items. None of the participants had access to all five objectively assessed qualities and the GIS based index score therefore ranged from 0 to 4.

### Area aggregations

The survey participants were grouped in area units of 1,000 square meters, resulting in 3,368 different areas. Areas with only one individual were accepted, and, in line with individual SGS, missing self-assessments of individual qualities were regarded as negative assessments. The proportion of positive assessments for each of the five green items was estimated in multilevel statistical models (one model for each item; see "Statistical analysis" below for details). Area-aggregated SGS was then calculated for each area as the sum of the five estimated proportions from the multilevel models. Each individual was assigned with the area-aggregated SGS of his/her living area in the analyses using area-aggregated scores.

### Validation

We validated the five items of the SGS separately as well as the index score, individually and area-aggregated. The validation was restricted to convergent validity, i.e. concordance with other measures of the same construct, and concurrent validity, i.e. associations with other constructs of relevance measured at the same time.

We assessed convergent validity of the SGS by investigating agreement and association with objectively assessed green qualities using GIS. Concurrent validity was assessed by i) investigating associations with a yes-no survey question concerning the perceived availability of a green open space (e.g. larger park or similar) or forest area within 5-10 minutes walking distance from the residence and ii) investigating association with a question regarding neighborhood satisfaction, assessed on a four-point agreement scale (see Additional file 2: appendix 2 for phrasing of these questions). To elucidate further differences between individual and area-aggregated self-reports regarding concurrent validity, we also investigated associations with vitality calculated as the median of four survey questions concerning feelings of being 'full of energy', 'full of life', 'not worn out' and 'not tired' from the 36-item Short-Form (SF-36) [30]. Neighborhood perception [19], self-reporting behavior and health [31] may differ considerably across population subgroups and we therefore carefully considered socio-demographic variables that could confound the associations and affect the convergent and concurrent validity (see next section).

### Statistical analysis

The proportions of positive assessments of the five green items in each area unit of size 1,000 square meters were estimated in multilevel ("ecometric") logistic regression models with two levels, individual and area [20]. One multilevel model was established for each of the five items separately with adjustments for socio-demographic variables i.e., sex, age, highest level of obtained education, economic difficulties, country of origin and type of residence (categorized as presented in table 1). The obtained area-level residual *U*_*k *_from each area *k *is given on a log odds scale and can thus be transformed to a proportion *P*_*k *_as

$$P_{K}=\frac{\exp\left(\alpha +U_{k}\right)}{1+\exp\left(\alpha +U_{k}\right)},$$

where α is the estimated overall (fixed) intercept of the logistic model and exp(*α*+*U*_*k*_) denotes *e *to the power of *α*+*U*_*k*_. By using area-level shrunken residuals to calculate proportions, areas with very few individuals obtain a proportion that is similar to the overall mean. Area-aggregated SGS was then calculated for each area unit as the sum of the five estimated proportions from the multilevel models. Convergent validity of SGS (individual and area-aggregated) versus the GIS-assessments 'as gold standard' were measured as correlation, using Spearman's rank correlation coefficient, as agreement, using the sum of sensitivity and specificity (for definitions see table 2) [32] and Cohen's kappa.

**Table 2:** Definitions of sensitivity and specificity [32].

	Objective assessments using GIS	
	**Absent**	**Present**

Self-assessments		
Absent	a0	a1

Present	b0	b1

Total	n0	n1

		

Sensitivity = True positive self-assessment = b1/n1		

Specificity = True negative self-assessment = a0/n0		

We assessed how convergent validity was affected by the socio-demographic variables (table 1) using ordinal regression analysis under the cumulative odds model with location parameters only [33]. This model estimates average odds ratios (OR) of all possible dichotomizations of the ordinal response variable, i.e. individual SGS. Ordinal regression with adjustment for the socio-demographic variables was also used to examine the concurrent validity of SGS (individual and area-aggregated) in relation to perceived availability of a green open space or forest area, neighborhood satisfaction and vitality. Estimated effects were compared with corresponding models where the GIS index score was used as measure of the green neighborhood environment. Results are presented for both unstandardized scores and for scores standardized by mean and standard deviation in order to make effect estimates more comparable. For area-aggregated SGS, we also compared estimated effects from single-level (individual level only) and multilevel (individual and area level) models.

All basic statistical analyses and single-level analyses were carried out using SPSS, version 15.0 for Windows (SPSS Inc, Chicago, Illinois, USA). Multilevel modeling was conducted in MLwiN version 2.19 (Centre for Multilevel Modeling, University of Bristol, U.K.).

## Results

### Convergent validity

**Figure 1:**
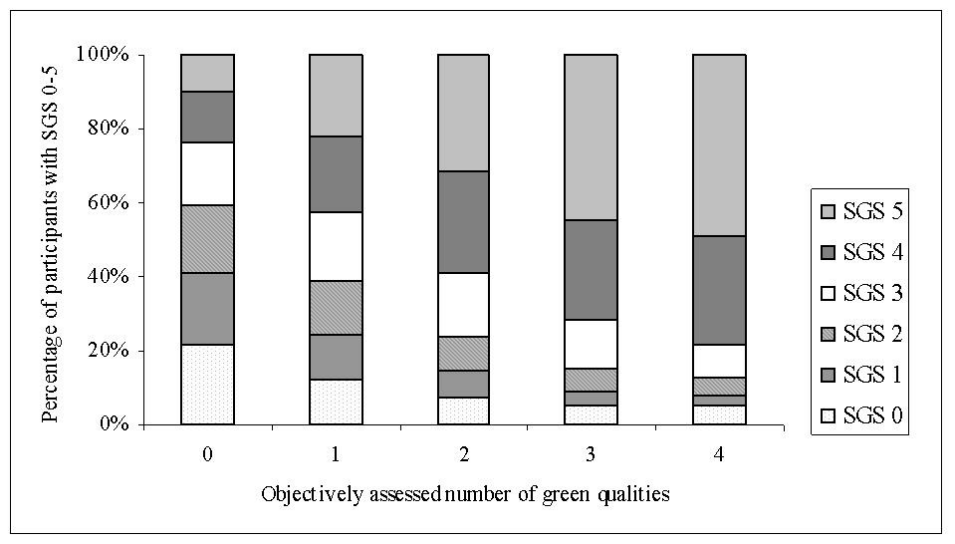
**SGS and objectively assessed green qualities**. The Scania Green Score in relation to the objectively assessed number of green neighborhood qualities. None of the participants had access to all five qualities as measured with GIS.

The individual SGS and the corresponding sum of the objective assessments were clearly correlated (Spearman's rank correlation = 0.35; N = 24,847; figure 1). This correlation was even more evident when the area-aggregated SGS was used (Spearman's rank correlation = 0.51; N = 24,582). The correlation between the self-reported and objectively assessed individual qualities was clear for Lush (Spearman's rank correlation = 0.32; N = 22,121) but less evident for Culture, Serene, Spacious and Wild (Spearman's rank correlation range = 0.15-0.22; N range = 21,499-23,146).

Perceived availability was higher than objectively assessed availability for all green neighborhood qualities (table 3). Agreement between self-reports and objective assessments, measured as sum of sensitivity and specificity was generally low for all five qualities when compared individually. The overall agreement did not differ noticeably depending on distance from residence covered by the objective assessments (100, 300 or 500 meter). Cohen's kappa ranged between 0.03 and 0.27 for the five individual items of SGS (not in tables). The agreement between the two index scores on the individual-level was also low (Kappa = 0.02; N = 24,848), with higher score on average in individual SGS than in the GIS index score (mean = 2.58; SD = 1.73; N = 25,029 versus mean = 0.71; SD = 1.04; N = 25,847).

**Table 3:** Agreement between the individual-level self-assessed (< 5-10 min walking distance) and objectively assessed availability (< 100, 300 and 500 meters distance from residence) of the five green qualities, Culture, Lush, Serene, Spacious and Wild. Agreement is given as sensitivity and specificity (%), separately and as a sum.

	Prevalence (%)		Agreement (%) ^a^									
Green quality			N	100 m			300 m			500 m		

	GIS	Self		Sum	Sens	Spec	Sum	Sens	Spec	Sum	Sens	Spec

Culture	22	39	21,499	123	65	58	120	60	60	117	57	61

Lush	27	51	22,121	136	90	46	135	82	53	136	77	59

Serene	7	73	23,146	119	97	22	119	96	22	119	96	23

Spacious	11	64	22,551	121	90	31	120	88	32	121	87	33

Wild	4	31	22,383	137	71	66	134	67	67	131	62	69

^a ^Sensitivity was defined as the proportion of "true" positive self-assessment and the specificity as the "true" negative self-assessment, using the objective assessments based on GIS as gold standard. Missing self-assessments and don't know scores were excluded.

**Figure 2:**
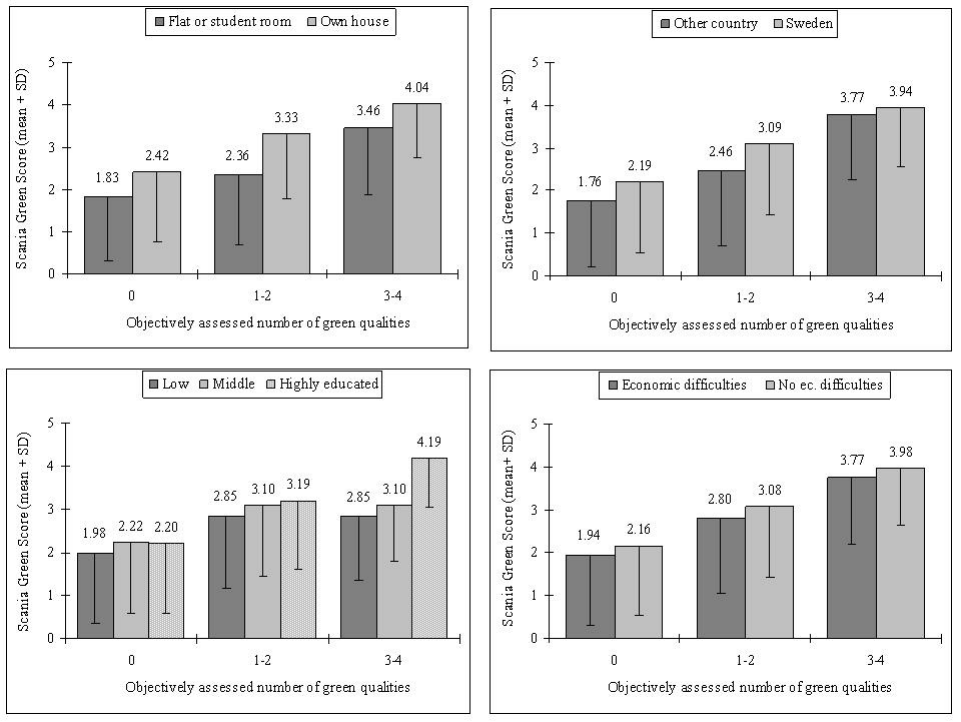
**SGS for various socio-demographic subgroups**. The mean Scania Green Score with standard deviation for various socio-demographic subgroups, stratified for objectively assessed number of green neighborhood qualities.

The individual SGS, given a certain number of objectively assessed green qualities in the near neighborhood, was markedly higher especially among house-owners, but also among participants born in Sweden and among the highly educated (figure 2). SGS also seemed higher among participants without economic difficulties but this association did not remain after adjustment for the other socio-demographic characteristics (table 4). The score was somewhat higher among middle-aged, whereas no difference in SGS was found between males and females.

**Table 4:** Differences for various socio-demographic subgroups in Scania Green Score (SGS), the index score of self-assessed availability of five green qualities within 5-10 minutes walking distance from the residence.

Subgroup		OR	(95% CI)
Sex			

	Females	1.00	

	Males	1.02	(0.97-1.07)

Age			

	18-34	1.00	

	35-49	1.25	(1.16-1.34)

	50-64	1.17	(1.09-1.25)

	65-80	0.89	(0.82-0.96)

Educational level			

	Primary and/or secondary school	1.00	

	2-4 years gymnasium or professional school	1.16	(1.09-1.24)

	University	1.19	(1.12-1.28)

Economic difficulties			

	Yes, at least every second month	1.00	

	No, never or once	1.06	(0.97-1.17)

Country of origin			

	Other	1.00	

	Sweden	1.42	(1.32-1.52)

Type of residence			

	Flat or student room	1.00	

	Own house	1.94	(1.84-2.04)

The first category of each socio-demographic variable is the reference. Higher odds ratios indicate a higher SGS. The multiple ordinal regression model was adjusted for the objectively (GIS-based) assessed number of green neighborhood qualities.

### Concurrent validity

Perceived availability of a green open space (or forest area) within 5-10 minutes walking distance from the residence was clearly associated with the individual SGS. Participants who did not perceive a green open space in their near neighborhood on average had a SGS of 1.19 qualities (SD = 1.46; N = 1,498) while participants who did perceive a green open space in the near neighborhood had a SGS of 2.72 qualities (SD = 1.68; N = 22,834) on average. The association remained strong when adjusted for socio-demographic variables in an ordinal regression model (OR = 6.7; 95% CI = 6.0-7.4; N = 21,632). Availability of a green open space was also associated in ordinal regression with the area-aggregated SGS rounded to the nearest integer (OR = 3.1; 95% CI = 2.8-3.5) and with the index score of the objective assessments (OR = 2.0; 95% CI = 1.8-2.2).

The index scores were clearly associated with neighborhood satisfaction, also after adjustment for socio-demographic variables (table 5). Focusing on the standardized scores, the association with neighborhood satisfaction was strongest for individual SGS and similar, but weaker for area-aggregated SGS and the GIS index score. Only individual SGS was clearly associated with self-rated vitality. Effect estimates with confidence intervals associated with area-aggregated SGS were similar in single-level and multilevel models, suggesting limited clustering remaining in the outcome variables within areas after covariate adjustments.

**Table 5:** The association between individual and area-aggregated (1,000 square meter areas) self-assessments of Scania Green Score (SGS), objective GIS-based assessments of number of green neighborhood qualities and neighborhood satisfaction (N = 21,665) and vitality (N = 20,855).

		Neighborhood satisfaction		Vitality	
**Model**		**OR**	**95% CI**	**OR**	**95% CI**

Single-level	**Unstandardized scores**				

	SGS individual	1.30	(1.28-1.32)	1.08	(1.06-1.10)

	SGS area-aggregated	1.48	(1.42-1.56)	1.04	(1.00-1.08)

	GIS index score	1.19	(1.16-1.23)	0.99	(0.97-1.02)

	**Standardized scores**				

	SGS individual	1.58	(1.53-1.63)	1.14	(1.11-1.17)

	SGS area-aggregated	1.30	(1.26-1.34)	1.03	(1.00-1.05)

	GIS index score	1.30	(1.16-1.24)	0.99	(0.97-1.02)

Multilevel	**Unstandardized scores**				

	SGS area-aggregated	1.51	(1.42-1.61)	1.04	(1.00-1.09)

	**Standardized scores**				

	SGS area-aggregated	1.32	(1.26-1.38)	1.03	(1.00-1.06)

Effect estimates are per unit increase in the index scores and the ordinal regression model was adjusted for sex, age, educational level, economic difficulties, country of origin and type of residence. Results are presented for both unstandardized and standardized (by mean and standard deviation) scores. For area-aggregated SGS results are presented both for single-level and multilevel models.

In an effort to separate effects of perceived and objectively assessed scores, we added GIS index score to the single-level models for SGS (individual/area-aggregated) and neighborhood satisfaction. In those models, the effect estimates of the (aggregated) SGS decreased only marginally (results not presented), whereas the effect estimate of the standardized GIS index score became markedly lower (e.g. OR = 1.08, 95% CI 1.04 - 1.12 in model for area-aggregated SGS and neighborhood satisfaction; not in tables). Similarly, the effect estimates for neighborhood satisfaction associated with SGS decreased marginally also when perceived availability of a green open space or forest area (yes/no) was added to the models (e.g. OR associated with standardized area-aggregated SGS decreased from 1.30 to 1.27; not in tables).

## Discussion

### Principal findings

SGS exhibited convergent validity through its clear association with objective measures of green neighborhood qualities, although the agreement was low. Concurrent validity was also demonstrated, but it should be stressed that the association between individual SGS and vitality could not be replicated when objective measures of the neighborhood were used. In contrast, area-aggregated SGS yielded associations consistent with the objective measures and may therefore be a useful approach to avoid bias due to confounded self-assessments.

### Strengths and limitations of the study

To the best of our knowledge, this is one of the first studies assessing validity of individual as well as area-aggregated self-reports of green neighborhood qualities in relation to objective assessments and concurrent health questions in a large and representative sample with detailed adjustment for socio-demographic variables. The multilevel (ecometric) analysis takes the varying sampling size across areas into account and also facilitates adjustment for confounding from socio-demographic factors in the self-reports. Another strength of the study was the focus in SGS on perceived qualities of the green neighborhood. Our results suggest that perceived qualities are likely to be relevant for health and well-being in addition to more simple constructs such as perceived availability of a green open space or forest area. An attractive feature of the area-aggregated SGS is that it captures perception of the green environment while being stronger correlated with objective measures and less susceptible to single-source bias compared to the individual self-reports.

An important limitation of the study was that we were not able to validate self-reports in the most urbanized inner city areas. The perception and the relative importance of green neighborhood qualities on health may very well be different in inner city areas. Furthermore, the most urbanized city areas are likely to accommodate large groups of individuals who could be more dependent on the (green) neighborhood environment they live in, e.g. people who spend a larger amount of their time at home [3] and tenants, who often lack access to an own garden [12].

Another limitation was the cross-sectional study design that limited the ability to assess temporal associations, i.e. predictive validity of the SGS [34,35]. The low agreement with the self-assessments of green qualities, and the relatively weak association between GIS index score and neighborhood satisfaction after including SGS in the model, may also raise concerns about the GIS-based assessments regarded as gold standard in our study. These objective assessments, developed by experts in landscape planning, show clear associations with neighborhood satisfaction and physical activity [6], but are not validated constructs. Main points of concern are i) the data sources reflect physical attributes, e.g. land use, while originally the definitions of the qualities are based on individual preferences, and ii) the assessments may suffer from inaccuracies and lack of sufficient detail in the land cover classification [29].

In order to limit the number of analyses we restricted the use of area-aggregated measures to the index score (SGS). With equal weights for all five qualities in SGS, the inherent assumption is that the qualities are all equally important for the health indicators under investigation (i.e., more qualities is always better for health), but this assumption can of course be questioned. The green qualities are distinct entities, e.g. wild environments (plants seem self-sown, lichen and moss-grown rocks, old paths etc.; see Additional file 1: appendix 1) are clearly different from e.g. environments rich in culture (a historical place offering fascination with the course of time; historical sights and remains etc.). Identification of specific elements (aspects, qualities) in natural environments that promote human health is an issue of great interest currently within landscape planning and environmental health [16,20,27]. In the present paper, we calculated an area-aggregated proportion for each quality in a two-level model (individual and area) rather than in a three-level model (item, individual and area; [20]). Such a two-level model can be used in future studies to assess which attributes are most important for health and well-being. Our previous work on the qualities included in the SGS suggests that these qualities may not be equally associated with health indicators such as neighborhood satisfaction and physical activity [12].

One could argue about the choice to regard missing self-assessments as negative (counted as zero in SGS) and about the choice to use areas of 1,000 square meters. However, associations of the (aggregated) SGS with neighborhood satisfaction and vitality remained similar when individuals with missing assessments were excluded (results not presented). Secondly, a sensitivity analysis showed that the 1,000 square meter assessments correlated strongly with the 500 (Spearman's rank correlation = 0.91; N = 24,480) and the 2,000 square meter assessments (Spearman's rank correlation = 0.92; N = 24,636; not in results). Estimates from the ordinal regression model also remained similar when we used self-assessments aggregated to 500 and 2,000 meter areas (results not shown). Though, the boundaries of our grids may not correspond with the boundaries that delimited the true collective (e.g. neighborhood) that influences individual health [36].

### The results in relation to other studies

The correlations between objectively assessed and self-reported green qualities may seem weak (Spearman's rank correlation range = 0.15-0.32) but are in line with correlations found in cross-sectional settings where exposure-response associations indeed are strong (e.g. correlation between GIS modeled residential road noise and self-reported annoyance, Spearman's rank correlation r = 0.20 [37]).

A recent report showed that the perceived environment correlated stronger with adolescents' physical activity behavior than the objectively assessed environment [22]. In our study, the association between the original (unstandardized) GIS-index score and neighborhood satisfaction was similar to the association with individual SGS while the association with area-aggregated SGS was more pronounced. However, this can be explained by different scaling and spread in the assessments; low spread tends to inflate while high spread tends to decrease the odds ratios. When we used standardized measures to compare the three scores we indeed found a more pronounced effect of individual SGS and similar effects of area-aggregated SGS and the GIS-based index score. The effect of the GIS-based index score decreased markedly when area-aggregated SGS was included in the model, which could suggest that perceived attributes of the green environment are more important for neighborhood satisfaction. However, alternative explanations for this finding such as differences in spatial resolution between the GIS-based index score (300 meter from the individual residences) and area-aggregated SGS (residences aggregated in 1,000 square meter areas) cannot be ruled out.

Results for vitality did suggest confounding (single-source bias) since a clear association was present for individual SGS only. This bias was most likely caused by individual characteristics affecting self-reporting behavior which were not fully captured by the included socio-demographic factors. How self-reports aggregated to narrow area units might decrease bias from unmeasured determinants of self-reporting behavior has been demonstrated by simulations [38]. Aggregated self-reports have been used as exposure measure in practice when monitoring or assessing health effects of e.g., neighborhood characteristics (e.g. resources for physical activity, safety, crime, dissatisfaction with green space, availability of parks) [20,22,25,39], air pollution [40-42], traffic noise [43], and job strain [44].

Agreement between perceived and objectively assessed availability of the individual green neighborhood qualities was low and comparable to previous studies [22,45]. However, our study looked at specificity and sensitivity as separate measures of agreement. The sensitivity of the self-reports was generally satisfactory whereas the specificity was low, implying that the perceived availability of green neighborhood qualities within 5-10 minutes walking distance was considerably higher than objectively assessed availability within 300 meters from the residence. One explanation for the low agreement could be that what is perceived as "5-10 minutes walking distance" may vary extensively among study subjects. However, changing distance from 300 to 100 or 500 meters in the GIS-based assessments did not increase agreement noticeably. Another explanation for the low agreement could be that the definitions used for the GIS-assessments were more extensive, and consequently more restrictive, than the phrasings used in the survey questions.

Socio-demographic factors were associated with the number of perceived green qualities in the neighborhood, which might also contribute to the low agreement. Such associations have also been demonstrated for self-reports of neighborhood attractiveness and safety [19] and other environmental factors [37]. Negative perceptions could be related to factors found more prevalent in groups with low compared to high socio-economic status, i.e. low social capital, poor health and a more pessimistic world view, but could also be due to the possibility that objective measures not necessary capture all environmental attributes that participants take into account in their perceptions [19]. Compared to the individual self-reports, the area-aggregations were stronger correlated with the GIS-based assessments, indicating lower amount of confounding and/or random misclassification error.

### Implications for further research

Assessing the green qualities on an ordinal rather than binary scale using GIS, also in urban areas, would facilitate a more detailed validation of the self-reported items and would provide opportunities for index scores with wider ranges. Qualities of neighborhood green space in relation to health outcomes merits further investigation in longitudinal settings.

## Conclusions

Perceived and objectively assessed qualities of the green neighborhood environment correlate but do not agree. Our study shows that an index score based on self-reports aggregated to narrow area units can be a valid and useful approach for assessments of perceived neighborhood qualities in settings where objective assessments are not possible or feasible. An area-aggregated index score like our SGS could then be used in health-related environmental monitoring, prospective epidemiological research and development of healthy living environments.

## List of abbreviations

GIS: Geographical information system; SGS: Scania Green Score (a new index score comprising five green qualities); SF-36: 36 item short-form (used to measure vitality); OR: Odds ratio; 95% CI: 95% confidence interval.

## Competing interests

The authors declare that they have no competing interests.

## Authors' contributions

All authors have directly participated either in the planning, execution, or analysis of this study and have read and approved the final version submitted. The study was initiated by MA and JB and further developed by KJ, MA and JB. The statistical analysis was carried out by KJ and JB in cooperation with MA and JM. All authors have revised drafts and contributed to the discussion.

## Supplementary Material

Additional file 1 **Appendix 1. Original descriptions of the five green qualities and GIS inclusion criteria **a. Original descriptions of the five green qualities of the neighborhood environment that are comprised into the Scania Green Score. b. Inclusion criteria as used in the objective neighborhood analyses (with GIS) to measure availability of each of the five green qualities. Click here for file

Additional file 2 **Appendix 2. Survey questions **Survey questions concerning green neighborhood qualities, neighborhood satisfaction and vitality as formulated in the extensive general health questionnaire conducted in Southern Sweden in 2008 which was used for this study (translated from Swedish). Click here for file
