# Supplementary material for: Area-aggregated assessments of perceived environmental attributes may overcome single-source bias in studies of green environments and health: results from a cross-sectional survey in southern Sweden
Source: Environ Health. 2011 Jan 17;10:4. doi: 10.1186/1476-069X-10-4 (PMC3032647; doi:10.1186/1476-069X-10-4)
Supplement: Additional file 1 — Appendix 1. Original descriptions of the five green qualities and GIS inclusion criteria a. Original descriptions of the five green qualities of the neighborhood environment that are comprised into the Scania Green Score. b. Inclusion criteria as used in the objective neighborhood analyses (with GIS) to measure availability of each of the five green qualities. [file 1476-069X-10-4-S1.DOC]

| **Quality** | **Description** |
| --- | --- |
| Culture | The essence of human culture. A historical place offering fascination with the course of time |
| Lush | A place rich in species. A room offering a variety of wild species of animals and plants |
| Serene | A place of peace, silence and care. Sounds of wind, water, birds and insects. No rubbish, no weeds, no disturbing people. |
| Spacious | A room offering a restful feeling of “entering another world”, a coherent whole, like a beech forest. |
| Wild | A place of fascination with wild nature. Plants seem self-sown. Lichen and moss-grown rocks, old paths. |

**Appendix 1. Original descriptions of the five green qualities and following GIS inclusion criteria**

a. Original descriptions of the five green qualities of the neighborhood environment that are comprised into the Scania Green Score.

b. Inclusion criteria as used in the objective neighborhood analyses (with GIS) to measure availability of each of the five green qualities.

| **Quality:** | **Serene** | **Wild**^a^ | **Lush** | **Space** | **Culture** |
| --- | --- | --- | --- | --- | --- |
| Data source | Included areas | | | | |
| CORINE land cover nomenclature, EU[[1]](#endnote-2) | 3.1.1 Broad-leaved forest  3.1.3 Mixed forest  4.1.1 Inland marshes  4.1.2 Peatbogs  5.1 Inland waters | 3.1. Forests  3.2.4.1 Transitional woodland shrub  3.3.2 Bare rock  4.1. Inland wetlands  5.1. Inland waters  They must be >15 ha, or be closer to urban area than 1 kilometre | 3.1.3 Mixed forest  3.3. Open spaces with little or no vegetation  4. Wetlands | 3.1 Forests >25 ha  3.2.1. Natural grassland  3.2.2. Moors and heathland  3.3. Open spaces with little or no vegetation  4. Wetlands | 1.4.2.5 Non-urban parks |
| Topography, |  | Slopes > 10 degrees | All registered “key biotopes” | Slopes > 10 degrees | Farmland to protect, pointed out in a national plan |
| Certain regional inventories |  |  | Pasture land of regional interest | Farmland to protect, pointed out in an national plan | National interests of cultural preservation |
| administrated by the County Administration |  |  | Biodiversity areas, Bird biotopes ref. Nature 2000 | Coastal zones preservation | Nature reservation areas |
|  |  |  | National park |  |  |
|  | Excluded areas | | | | |
| A regional noise inventory, County Adm | Noise > 30 dB(A) | Noise > 40 dB(A) |  | Noise > 40 dB(A) |  |
|  | Artillery range | < 800 m distance to wind power aggregates |  |  |  |

^a^ All included areas for the Wild characteristics must either be located less than 1 km from a village or have a size above 15 ha

1. CORINE land cover nomenclature, 1994, Part one - Chapter 2: Basic principles, CORINE land cover 21, Table 2.2. European Environment Agency, an agency of the European Union.  [↑](#endnote-ref-2)
