# Supplementary material for: Area-aggregated assessments of perceived environmental attributes may overcome single-source bias in studies of green environments and health: results from a cross-sectional survey in southern Sweden
Source: Environ Health. 2011 Jan 17;10:4. doi: 10.1186/1476-069X-10-4 (PMC3032647; doi:10.1186/1476-069X-10-4)
Supplement: Additional file 2 — Appendix 2. Survey questions Survey questions concerning green neighborhood qualities, neighborhood satisfaction and vitality as formulated in the extensive general health questionnaire conducted in Southern Sweden in 2008 which was used for this study (translated from Swedish). [file 1476-069X-10-4-S2.DOC]

## Appendix 2. Survey questions

**Green qualities**

- Think of nature within 5-10 minutes walking distance from where you live. For example this can be green spaces, parks or forest areas. Do you agree with the following statements?

*Choose an option from each line!*

Nature in the area where I live *Totally Totally Do not know/*

…….  *not agreeing Not agreeing Agreeing agreeing Can not say*

1. .. is quiet, one can hear nature’s

own sound ^*1^

1. .. is wild, it has developed without

without human impact ^*1^

1. .. has a large diversity of animal

and plant species ^*1^

1. .. is a large cohesive area ^*1^
2. .. makes you feel the historical

heritage, for example ancient

monuments, old trees, constructions ^*1^

^*1^ Note: In which a. represents *serene*; b. *wild*; c. *lush*; d. *spacious*; e. *culture*.

**Availability of a green open space or forest area**

- Is there a green open space (large park or comparable) or forest area within 5-10 minutes walking distance from where you live?

Yes

No

I don’t know

**Neighborhood satisfaction**

- What is it like living in your neighborhood?

Very good

Rather good

Rather bad

Very bad

Do not know/ not relevant

**Vitality**

- In the last four weeks, how often did you feel *full of life, full of energy, worn out, tired*? ^*2^

*All the time largest part large part partly rarely never*

1 2 3 4 5 6

*Full of life*

*Full of energy*

*Worn out*

*Tired*

^*2^ Worn out and tired scores are inversed before taking the mean of the four questions.
